# Interaction of Polybrominated Diphenyl Ethers and Aerobic Granular Sludge: Biosorption and Microbial Degradation

**DOI:** 10.1155/2014/274620

**Published:** 2014-05-29

**Authors:** Shou-Qing Ni, Qingjie Cui, Zhen Zheng

**Affiliations:** ^1^Shandong Provincial Key Laboratory of Water Pollution Control and Resource Reuse, School of Environmental Science and Engineering, Shandong University, Jinan 250100, China; ^2^Department of Mechanical and Environmental Protection, Shandong Electric Power Engineering Consulting Institute (SDEPCI), Jinan 250013, China

## Abstract

As a new category of persistent organic pollutants, polybrominated diphenyl ethers (PBDEs) have become ubiquitous global environmental contaminants. No literature is available on the aerobic biotransformation of decabromodiphenyl ether (BDE-209). Herein, we investigated the interaction of PBDEs with aerobic granular sludge. The results show that the removal of BDE-209 from wastewater is mainly via biosorption onto aerobic granular sludge. The uptake capacity increased when temperature, contact time, and sludge dosage increased or solution pH dropped. Ionic strength had a negative influence on BDE-209 adsorption. The modified pseudo first-order kinetic model was appropriate to describe the adsorption kinetics. Microbial debromination of BDE-209 did not occur during the first 30 days of operation. Further study found that aerobic microbial degradation of 4,4^′^-dibromodiphenyl ether happened with the production of lower BDE congeners.

## 1. Introduction

Polybrominated diphenyl ethers (PBDEs), a class of persistent organic pollutants (POPs) and formerly widely used flame retardants in manufactured materials, are found in different environmental media and have become ubiquitous global environmental contaminants. The mixtures of pentabromodiphenyl ether (penta-BDE), octabromodiphenyl ether (octa-BDE), and decabromodiphenyl ether (deca-BDE, BDE-209) were the most common commercial products till May 2009 when the penta- and octa-BDEs were added to the list of new POPs by the parties of the Stockholm Convention for POPs [1]. It should be noted that penta-BDE was exclusively used in the US till 2004 when penta- and octa-BDEs were voluntarily removed from production. Therefore, BDE-209 is the only remaining commercial PBDE product used widely in large quantities in the global market [2]. Literature had concluded that deca-BDE could be transformed into more toxic lower brominated congeners in the environment [2, 3], representing a significant source of new POPs.

Wastewater treatment plants (WWTPs) collect different types of wastewaters and play important roles in waste disposal. In the process WWTPs become receivers of POPs such as PBDEs. US EPA Toxic Release Inventory (TRI) revealed that in the US over 500 metric tons (MT) BDE-209 per year were discharged to land via on- and off-site industrial releases from 1988 to 2004 [4]. Among these more than 90 MT per year were transferred to WWTPs. The EPA TRI data show that in 2009 there were 509,839 pounds of BDE-209 released in the US due to the voluntary reduction of usage. Song et al. [5] showed that the total loading of *∑*_5_ PBDE (sum of BDEs 47, 99, 100, 153, and 154, five main compositions of commercial penta-BDEs) to the Little River, leading to the Detroit River, from a WWTP was about 1900 mg/d or 0.7 kg/year, indicating that the discharges from WWTP are a major contamination source of PBDEs in receiving water bodies. Concentrations of PBDEs were detected from 265 to 51,232 ng/L and from 26 to 39 ng/L in the raw wastewater and the treated effluent from WWTPs in North America, meaning that about 10% of PBDEs input was discharged into receiving water bodies [5–7]. Consequently, BDE-209 was reported to be the predominant congener in the river water and sediments [8]. Significant concentrations of PBDEs were also detected in wastewater treatment sludge. Hale et al. [9] found BDE-209 concentrations varied from 84.8 to 4890 *μ*g/kg among different disposed sewage sludge. They also discovered that BDE-209 was one of the main PBDEs in the land-applied sludge. In China, as high as 34900 *μ*g BDE-209/kg sewage sludge was detected from the largest WWTP in Shanghai [10]. Obviously, sewage sludge samples contained higher BDE-209 level from Asia than those from North America and Europe [9–11]. The widespread detection of PBDEs in WWTP sludge implies that land application of these biosolids can contribute PBDEs to terrestrial systems, that is, increasing PBDEs soil concentrations when applied on agricultural land.

As wastewater passed through the WWTP, the concentrations of PBDEs tended to decrease, while these in sludge had a tendency to concentrate [5]. It is of importance to evaluate the interaction of PBDEs with wastewater sludge. To date, limited research has examined the fate and transport of the current widely used BDE-209 in wastewater granular sludge. Aerobic granular sludge is an important type of granular sludge that provides many advantages over the conventional activated sludge. These include dense microbial structure, good settling property, high biomass concentration, and good capacity to deal with toxic medium [12]. At present, aerobic granulation technologies have been developed for treating various types of high strength wastewaters containing organics, nitrogen, phosphorus and toxic substances, and so forth [13]. Aerobic granules, having large surface area and high porosity, are considered excellent biosorbents for dyestuff and heavy metal removal [13, 14]. It has been estimated that over 90% of the PBDEs entering a WWTP ultimately concentrated within the sludge, with the remainder released via its effluent [5]. However, little knowledge is available regarding the biointeraction of PBDEs with aerobic granules.

The objective of the present research was to examine the abiotic accumulation and biodegradation of PBDEs, a model POP, by aerobic granules, which is essential to evaluate the safety of biosolids land application. The biosorption properties were explored as a function of batch operating conditions including solution pH, contact time, sludge dosage, initial PBDE concentration, ionic strength, and temperature. Kinetic and isotherm analysis of the biosorption process was performed. The possible microbial degradation of fully brominated BDE-209 and lower BDE congeners, using 4,4′-dibromodiphenyl ether (BDE-15) as a model, was also studied.

## 2. Materials and Methods

### 2.1. Materials

**Figure 1:**
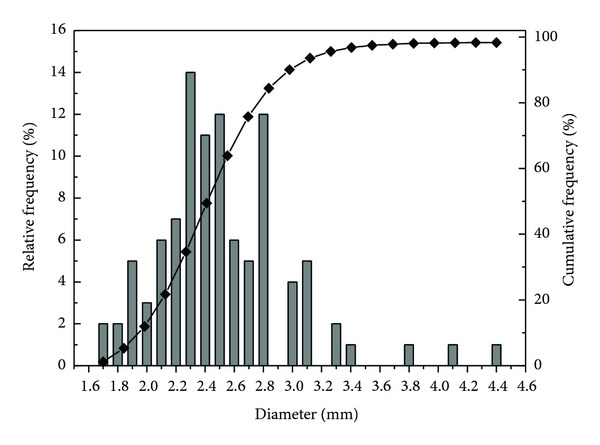
Granule size distribution and cumulative frequency (◆) of aerobic granules in the reactor.

A sequencing batch airlift reactor (SBAR) with a working volume of 3.5 L was stably operated at room temperature with acetate and glucose as the substrate at an organic loading rate of 500 mg COD/L. The SBAR reactor was fed with synthetic wastewater buffered with baking soda. The synthetic wastewater also had enough trace elements to support bacterial growth [15]. Fresh aerobic granular sludge in this study was collected from the SBAR and used as the sludge source. Aerobic granules were lightly washed three times with deionised water before usage. The suspended solid (SS) and volatile suspended solid of the aerobic granular sludge were 102.0 g/L and 78.1 g/L, respectively. The mean granule size of aerobic granules in the reactor was 2.52 ± 0.31 mm (Figure 1).

The individual congeners of BDE-209 and BDE-15 were purchased from Tokyo Chemical Industry Co., Ltd. (Tokyo, Japan). By dissolving BDE-209 and BDE-15 in dimethyl sulfoxide (DMSO), the stock solution of PBDEs (500 mg/L) was obtained. The different initial concentrations of BDE-209 solution (1, 2, and 4 mg/L) were prepared by diluting the stock with the culture medium (containing 500 mg/L COD, 350 mg/L NH_4_^+^-N, and 1 mL/L mineral-salts medium). All PBDEs solution had the same DMSO concentration (2% of final concentration).

### 2.2. Experimental Process

To analyze the interaction of aerobic granular sludge with BDE-209, experiments were carried out by adjusting solution pH, ionic strength, sludge dosage, initial BDE-209 concentration, and temperature in a batch mode for a period of 24 hours. The aforementioned granular sludge was added to serum bottles containing required cultural medium at designed SS and BDE-209 concentrations.

To determine the effect of granular sludge dose on the interaction process, various amounts of biomass (2, 4, 6, and 8 g SS/L) were mixed with 4 mg/L BDE-209 solution at room temperature (20°C). Under the same incubation conditions, the effect of pH on the interaction process was investigated in the pH range from 2 to 11 at 4 g SS/L, 4 mg/L BDE-209, and room temperature. The effect of contact time and initial BDE-209 concentration was studied by agitating 4.0 g aerobic granular sludge in a series of serum bottles containing 1 L BDE-209 solution of known concentrations (1, 2, and 4 mg/L). Two milliliter samples were taken at designed time intervals. Equilibrium studies were carried out by adding 4.0 g SS/L of aerobic granular sludge in a series of serum bottles at four different temperatures (20, 30, 40, and 50°C). In order to assess the influence of ionic strength on the equilibrium uptake, different sodium chloride and magnesium chloride stock solutions were added to 4 mg/L BDE-209 solutions, containing 4 g SS/L granules, to make final contents of 0 to 0.5 mol/L at room temperature. All the adsorption experiments were shaken on a thermostatic rotary shaker at 150 rpm and carried out in duplicate. Besides, 500 mg/L COD, 350 mg/L NH_4_^+^-N, and mineral-salts medium were added in order to ensure the activities of microbes.

To examine the microbial biodegradation of BDE-209, 1 mg/L BDE-209 was pumped into the SBAR together with the synthetic wastewater. BDE-15 was added to further study the biodegradable ability of aerobic sludge. BDE-15 was added into 200 mL medium containing 1 g SS aerobic sludge at the concentration of 1.0 mg/L. Aliquots of the sludge were autoclaved to kill all microbes and used as negative controls. The 250 mL flasks were cotton-plugged and wrapped in aluminum foil. Samples were taken daily for extraction with dichloromethane and acetone/hexane (50/50, v/v) to test possible metabolic products.

### 2.3. Analysis

In order to identify the functional groups of the fresh and treated granular sludge on the surface, FTIR spectra were taken on a Bruker VERTEX-70 IR infrared spectrometer. The damp samples were freeze-dried before the analysis. FTIR spectra were measured on KBr pellets prepared by pressing mixtures of 1 mg dry powdered sample and 100 mg spectrometry grade KBr under a vacuum to avoid moisture uptake. High performance liquid chromatography (HPLC, P1201, Elite Analytical Instruments, Dalian, China) with a UV detector (UV 1201) and a Sinochrom ODS-BP C18 column (200 mm × 4.6 mm) was used to analyze BDE-209 concentrations. For analysis, aqueous samples were liquid/liquid extracted in dichloromethane and the collected extract was dried using anhydrous Na_2_SO_4_ (granular, 10–60 mesh, Fisher) and further concentrated down to 1 mL under a gentle N_2_ stream. HPLC-grade methanol was utilized as mobile phase and the rate was 1.0 mL/min. Quantification was performed with a standard curve of the BDE-209. Reaction products in the extracts were analyzed using a 7890A gas chromatography (GC) with a 5975C mass spectrometric (MS) detector (Agilent Technologies) equipped with a DB-5MS capillary column (15.0 m × 250 *μ*m × 0.25 *μ*m). One *μ*L of the sample was injected in splitless mode with the inlet maintained at 320°C. The oven was held initially at 90°C for 1 min and increased at the rate of 20°C/min to the final temperature of 320°C, which was held for 15 min. Helium was used as a carrier gas at 1.5 mL/min. The interface temperature for the MSD and the ion source temperature were both set at 230°C. The mass spectrometer was in selective ion monitoring (SIM) mode at an electron impact energy of 70 eV.

## 3. Results and Discussion

### 3.1. Effect of Contact Time and Initial BDE-209 Concentrations

**Figure 2:**
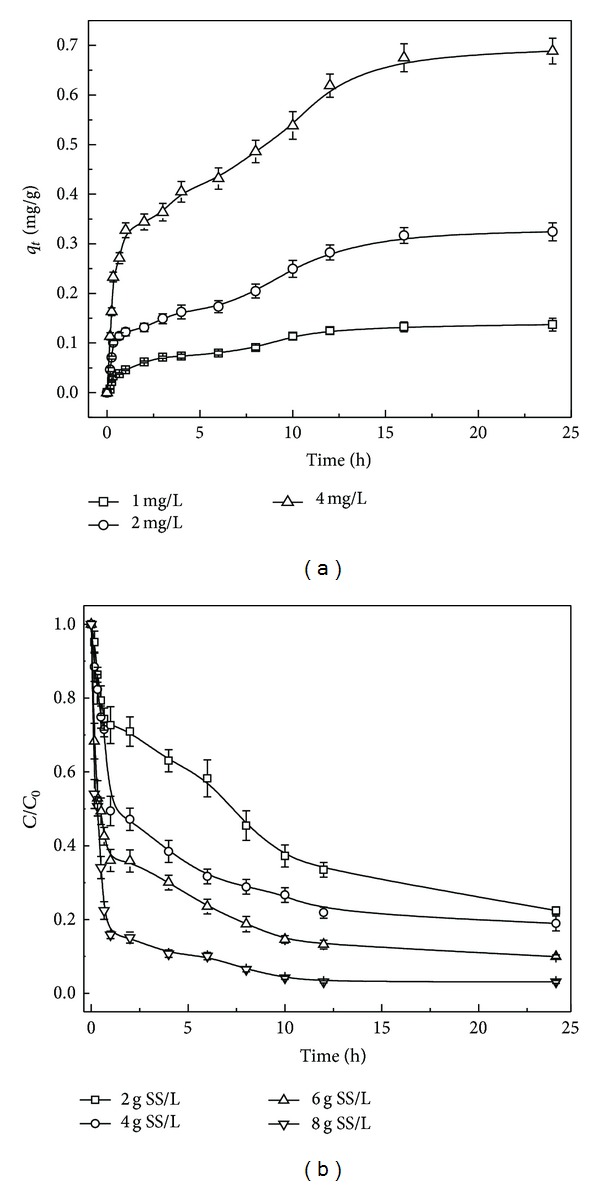
(a) Effect of contact time and initial BDE-209 concentrations on bioremoval of BDE-209 by aerobic granules. (b) Effect of different granular sludge dosages on biosorption process.

In order to measure the effect of initial BDE-209 concentrations, the sorption of BDE-209 onto aerobic granules was carried out at different initial concentrations.  Figure 2(a) shows the amount of BDE-209 adsorbed onto aerobic granules versus contact time at different initial concentrations. A rapid drop of BDE-209 concentration occurred at the beginning and then the BDE-209 amount absorbed gradually decreased with a longer period of time until equilibrium. After the equilibrium period, the amount of adsorbate nearly unchanged with time. The equilibration time was about 12 h. On changing the initial concentration, the amount of BDE-209 adsorbed increased from 0.14 to 0.69 mg/g SS.

The initial concentration gives an important driving force to overcome all mass transfer resistances of BDE-209 between the aqueous and solid phases. Consequently, a higher initial BDE-209 concentration would enhance the adsorption process. Moreover, the adsorption amount increased with the increase of initial BDE-209 concentration. The adsorption uptake capacity was high in the initial stage and generally conducted by the diffusion process from the bulk solution to the surface of aerobic granules [16]. After this phase, the adsorption may be an attachment-controlled process because of less available active sites. In conclusion, the effect of initial BDE-209 concentrations and contact time had been proved to be considerably important for the process of BDE-209 biosorption.

### 3.2. Effect of Aerobic Granule Dosages

To examine the aerobic granule dose effect, experiments were carried out with different granular sludge dosages at room temperature (Figure 2(b)). It was observed that removal rates for BDE-209 from the solution increased from 77.6% to over 95.0% when granular sludge dosages increased from 2 to 8 g SS/L. The microporous structure and relatively high surface area of aerobic granules assured the removal of BDE-209 from the solution. A very fast superficial adsorption onto sludge surface led to solute concentration in solution becoming lower when the aerobic granule doses became higher. Equilibrium uptake capacity decreased with increasing doses, though percentage of BDE-209 removed increased. Similar phenomenon was observed when using fly ash to remove PCBs from aqueous solutions [17]. Batch tests with activated sludge showed that the mass of PBDE compounds sorbed per unit mass of SS decreased with increasing SS concentrations as the available PBDEs were reduced and with higher initial concentrations the rate of PBDEs removal increased [18].

### 3.3. Effect of Initial Solution pH and Ionic Strength

**Figure 3:**
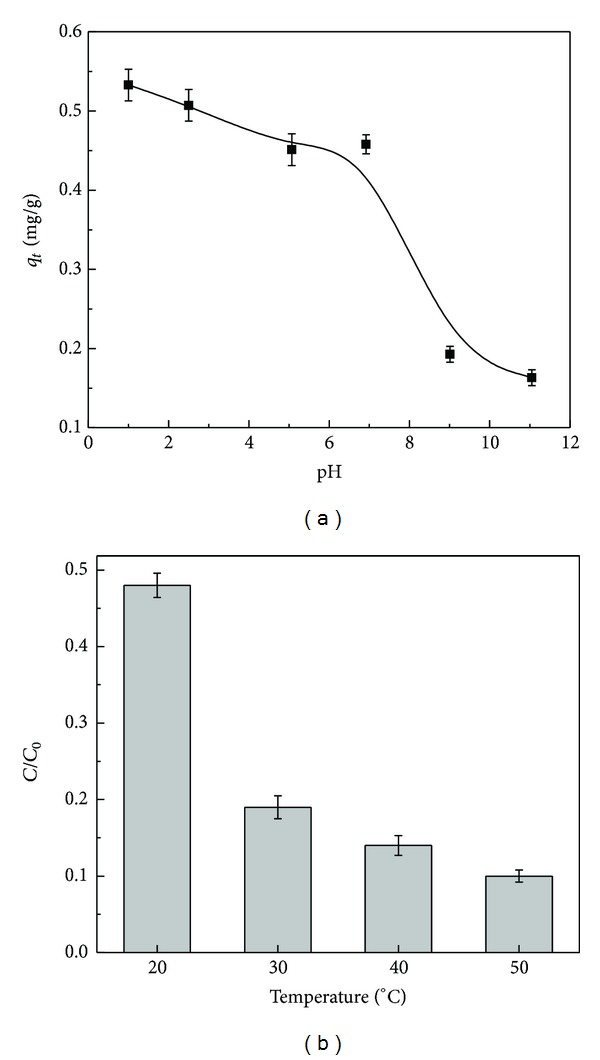
(a) Effect of initial solution pH on bioremoval of BDE-209 by aerobic granules. (b) Effect of different temperatures on bioremoval of BDE-209 by aerobic granules.

As the pH of initial solution is one of the important factors influencing the adsorption process, the removal amount of BDE-209 by aerobic granular sludge was studied at different pH values. The sorption procedure of BDE-209 on aerobic granular sludge was highly dependent on solution pH (Figure 3(a)). Equilibrium uptake capacity of BDE-209 by aerobic granular sludge decreased with the increase of pH in the range of 1–11. Due to the fact that solution pH influenced the surface charge of the adsorbent by the deprotonation and protonation of certain functional groups [19], the adsorption of PBDEs onto granules was affected. At acidic pH, the number of positively charged adsorbent sites increased. In this research, the point of zero charge of aerobic granular sludge was around pH 7.8, indicating that a zero net surface charge on the aerobic granular sludge was reached around pH 7.8. As shown in Figure 3(a), when the solution pH dropped below 7.0, biosorption capacity seriously decreased, proving that the surface charge of granular sludge or electrostatic interaction had an important role in this sorption. Langford et al. [18] revealed that less charged activated sludge resulted in increasing PBDEs partitioning. Residual BDE-209 in solution increased with increasing pH from 2.0 to 11.0 and this trend slowed down at pH values from 2.0 to 7.0 (Figure 3(a)).

Generally, there are various kinds of salts in wastewater leading to high ionic strength [20].  Table 1 suggested that the ionic strength of the solution negatively affected BDE-209 adsorption onto aerobic sludge. The removed amount of BDE-209 decreased when Na^+^ and Mg^2+^ concentrations in the solution increased from 0 to 0.5 M. This might relate to the 3-dimensional structures of the organic matters in granules. This might also be caused by the competitive binding for the sites available for adsorption, suggesting the nonselective nature of the adsorbent.

The effect of competitive binding increased with compound hydrophobicity [21]. With the highest log⁡*K*_ow_ value (distribution of a chemical between water and octanol, at equilibrium) among PBDE congeners, BDE-209 sorption rate was supposed to be easily affected. The PCBs removal on fly ash decreased when various salts, including NaCl, were introduced [17]. The binding sites for BDE-209 adsorption became fewer when the ionic strength increased [22]. It is shown in Table 1 that the sorption is also related to monovalent or bivalent ions. Bivalent electrolytes can provide more ions to ionic strength and more positive charge compared with univalent electrolytes. When ionic strength increased from 0 to 0.1 M, the influence of Mg^2+^ on adsorption was obvious. But the adsorption amount had little change with the increase of Na^+^ strength. Thus, the effect of Mg^2+^ on adsorption is more serious than Na^+^.

### 3.4. Effect of Temperature and Thermodynamic Parameters

The function of temperature in adsorption of BDE-209 onto granules is shown in Figure 3(b). It is obvious that the adsorption capacity increased with increasing temperature. This is similar to PCBs sorption by fly ash [17]. Based on the Arrhenius equation (S5, the relationship between temperature and rate constant), the activation energy (*E*_*a*_) was calculated as 45.2 kJ/mol through the slope. The thermodynamic parameters for this process were estimated using the following equations:

(1) $$\begin{array}{c} \Delta G^{0}=\Delta H^{0}-T\Delta S^{0}\\ \ln k_{C}=\frac{\Delta S^{0}}{R}-\frac{\Delta H^{0}}{\text{RT}}, \end{array}$$

where Δ*G* ^0^ (kJ/mol) represents the free energy change; Δ*H* ^0^ (kJ/mol) denotes the enthalpy change; Δ*S* ^0^  (kJ/(mol·K)) means the entropy change; *k*_*C*_ (L/g) is the standard thermodynamic equilibrium constant. The value of Δ*G* ^0^ is in the range of 7.1–9.8 kJ/mol. The values of Δ*H* ^0^ and Δ*S* ^0^ are calculated to be 31.4 kJ/mol and 0.12 kJ/(mol·K). The chemical (chemisorption) or physical adsorption (physisorption) mechanism is often an important indicator to describe the type of interactions between adsorbent and adsorbate. The adsorption capacity, which increased with increasing temperature, may be concluded to be chemisorption [23]. The magnitude of *E*_*a*_ gives an idea about the type of adsorption which is mainly physical or chemical. Low *E*_*a*_ of 5–40 kJ/mol is characteristic of physisorption, while high *E*_*a*_ of 40–800 kJ/mol suggests chemisorption [17]. In this study, *E*_*a*_ is in the range of chemisorption, confirming that the nature of BDE-209 adsorption by aerobic granules was a chemisorption process. Nollet et al. [17] indicated that the adsorption of PCB-173 was chemisorption (*E*_*a*_ = 49.08 kJ/mol) at room temperature. The positive value of Δ*H* ^0^ suggested that the chemisorption process was endothermic, similar to the results of Nollet et al. [17] and Hameed et al. [22]. The positive values of Δ*G* ^0^ implied the nonspontaneous nature of the process, while, with the increase of temperature, Δ*G* ^0^ decreased, indicating that the nonspontaneous nature of adsorption was inversely proportional to the temperature. The positive value of Δ*S* ^0^ suggested that increased randomness at the solid/solution interface occurred during the adsorption of PBDEs onto granules. The positive values of *E*_*a*_, Δ*G* ^0^ and Δ*H* ^0^ indicated the presence of an energy barrier in the adsorption process and endothermic process [22]. The positive values for these parameters are quite common because the activated complex in the transition state is in an excited form [23].

### 3.5. Adsorption Kinetics

**Figure 4:**
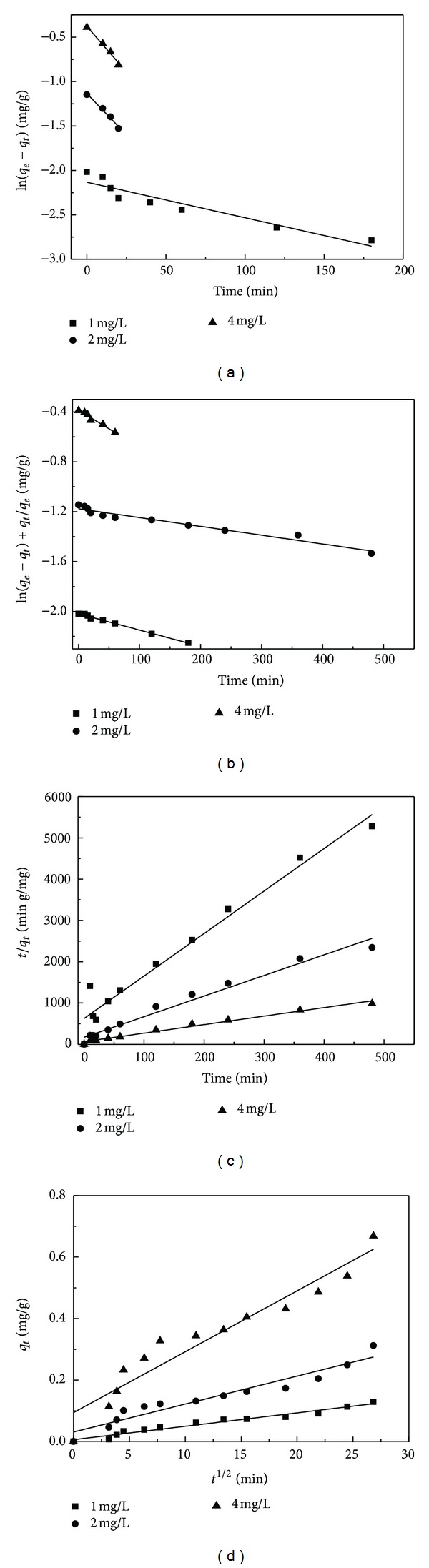
Kinetics plot for removal of BDE-209. (a) Pseudo first-order model, (b) modified first-order model, (c) pseudo second-order model, and (d) intraparticle kinetic model.

**Figure 5:**
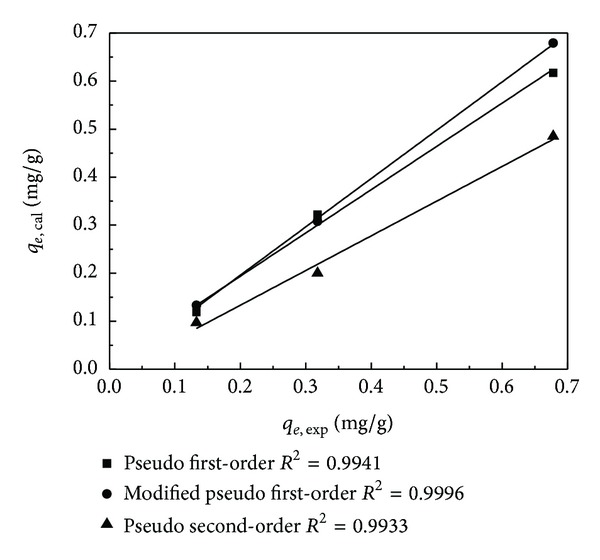
The plot of relationship between predicted adsorption quantity values and experimental values.

The adsorption kinetics of BDE-209 was analyzed to further understand the adsorption process of aerobic granular sludge. In this research, the dynamics of BDE-209 adsorption was analyzed using the pseudo first-order, the modified pseudo first-order, the pseudo second-order, and the intraparticle diffusion models (S1–S4, equations and explanation of above four models). As shown in Figure 4, the modified pseudo first-order model (Figure 4(b)) and the pseudo second-order model (Figure 4(c)) could describe the adsorption dynamics better than the pseudo first-order model (Figure 4(a)) and the intraparticle diffusion model (Figure 4(d)). The biosorption rates are faster than PCBs sorption using fly ash in comparison with *k*_1_ values [17]. The kinetic parameters for the adsorption of BDE-209 on aerobic granular sludge were listed in Table 2. The relationship between predicted adsorption quantity values and the experimental values is plotted in Figure 5. As it is shown in Table 2, the theoretical values of *q*_*e*_ calculated from the modified pseudo first-order kinetics fit well with the values obtained from the experiments (*R* ^2^ = 0.9996). Therefore, the modified pseudo first-order kinetic model was appropriate to describe the adsorption kinetics for the removal of BDE-209 onto aerobic granular sludge. The correlation coefficients of the modified pseudo first-order kinetic model were higher compared with other kinetic models. These suggested that the adsorption of BDE-209 onto aerobic granular sludge is probably controlled by chemical sorption. The intraparticle diffusion model has usually been the rate-limiting step but in our research it is not the only step because the intraparticle diffusion plot did not pass through the base point.

### 3.6. FTIR Analysis

**Figure 6:**
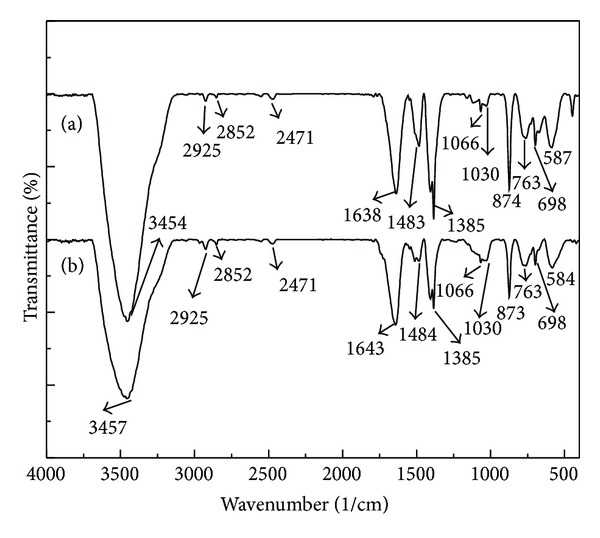
FTIR spectra of pristine (a) and BDE-209 treated (b) aerobic granules.

As we mentioned above, the electrostatic interactions played a very important role in BDE-209 adsorption on the aerobic granules because the electrostatic repulsion was the least around PZC (pH 7.8). However, it is interesting to note that the amount of adsorption decreased gradually when the solution pH increased from 1 to 8. It is therefore reasonable to assume that, in addition to the electrostatic interaction, other interactions may exist and play a role in the interaction of BDE-209 and aerobic granules. Taking into account the many functional groups on the surface of the granular sludge, one may expect that various interactions took place during bioremoval process. FTIR spectra have been a useful tool to identify the presence of certain functional groups in a molecule as each specific chemical bond often shows a unique energy band [24].  Figure 6 shows the typical FTIR results with the corresponding band assignments given in Table 3. The strong broad band ranging from 3200 to 3600 cm^−1^ usually corresponds to the possibility of overlapping between the –NH and the –OH stretching vibrations, and the strong sharp band at the wavenumber region of 3400–3550 cm^−1^ is characteristic of the –OH stretching vibration [24]. The significant decrease of transmittance in this band region after BDE-209 contact indicated that the –OH vibration was affected after the interaction. Other changes in the transmittances can be observed at the wavenumbers of 1638 and 1483 cm^−1^, corresponding to the C–N and N–H stretching, indicating that nitrogen atoms were the main interaction sites. Other major changes in the transmittance can also be observed at the wavenumbers of 1638, 1385, and 1030 cm^−1^; these band regions may be assigned to the C=O stretching, –COO^−^ stretching, and C–OH stretching [14, 25], confirming that oxygen atoms are highly involved in the interaction. Slight changes in the transmittance at the wavenumbers of 2925 and 2852 cm^−1^, related to the –CH_2_ stretching [14], were also observed. In agreement with XPS spectra (data not shown), oxygen and nitrogen atoms significantly contributed to BDE-209 binding, and the effect of carbon atoms appeared to be less important.

### 3.7. Microbial Biodegradation

PBDEs may be dehalogenated to lower brominated products in the environment [2, 26]. Several anaerobic species, such as* Dehalococcoides* species and* Dehalobacter* and* Desulfitobacterium* species, are responsible for the biotransformation of PBDEs [3, 27]. Besides pure cultures, mixed cultures also have the potential to debrominate PBDEs. Anaerobic bacterial mixed cultures isolated from river sediment and soil have been found to debrominate penta-through hexa-BDEs and hexa- through nona-BDEs, respectively [28, 29]. Bioremoval of PBDEs also occurred in a bioreactor and wastewater treatment plants [5, 7, 30]. However, few reports are available related to the aerobic debromination of PBDEs [31, 32]. Degradation of 4-monobrominated diphenyl ether to diphenyl ether was observed in aerobic sludge [31]. Generally, in biota highly brominated congeners first underwent anaerobic debromination, which was extremely slow [27], followed by fast aerobic degradation of less brominated congeners. BDE-209 was debrominated to hepta- and octa-BDEs by* Sulfurospirillum multivorans* culture after two months of incubation with trichloroethene [3]. Aerobic transformation of lower BDEs happened in hours [31, 32]. In this study, no lower brominated congeners were detected when BDE-209 was pumped into the airlift reactor during 30 days of operation as determined by GC-MS. This is predictable as aerobic microorganisms are only able to degrade lower BDEs. For example, two PCB degrading strains,* Rhodococcus jostii* RHA1 and* Burkholderia xenovorans* LB400, were able to transform the tested mono- through penta-BDEs and another two strains,* Rhodococcus *sp. RR1 and* Pseudonocardia dioxanivorans* CB1190, were only able to transform the less brominated mono- and di-BDE congeners [32]. To the best of our knowledge, no literature is available on the aerobic biotransformation of BDE-209.

**Figure 7:**
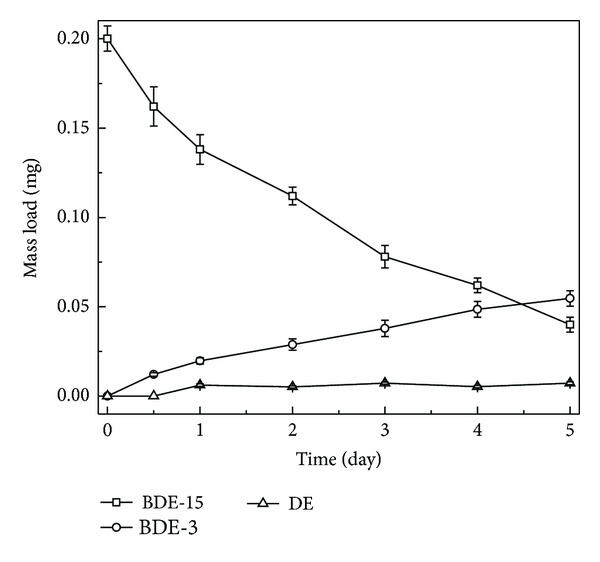
Microbial degradation of BDE-15 with the production of BDE-3 and DE.

To further study the biodegradable ability of aerobic sludge, BDE-15 was adopted as a model PBDE for low BDE congeners. We analyzed BDE-3 and DE generation concurrent with BDE-15 disappearance in order to confirm PBDE transformation.  Figure 7 shows the formation of BDE-3 and DE by aerobic sludge. Approximately 80% of added BDE-15 degraded with the production of 0.055 mg BDE-3 and 0.007 mg DE in 5 days. No biodegradation was observed in negative controls which were autoclaved to kill all microbes. We also found that bromide is released during PBDE transformation. Concurrent with the degradation of BDE-15, 60% of the stoichiometric bromide concentrations (theoretical bromide maximum) were recovered, indicating that specific bacteria in the granules are capable of cleaving the carbon-bromine bonds under aerobic conditions. BDE-15 was reductively debrominated in a fixed-film plug-flow biological reactor, leading to exclusive production of 4-bromodiphenyl ether (BDE-3) and diphenyl ether (DE) [30]. The lack of a complete mass balance (63–89% accounted for 5 days), together with the relative shortage of BDE-3 or DE, suggests that the debromination rate increased in moving as follows: BDE-15→BDE-3→DE. Consistent with anaerobic degradation [30], we found that the rate-limiting step is reduction of BDE-15→BDE-3. Then, BDE-3 readily debrominated to DE, which underwent rapid aerobic transformation to as yet unknown bioproducts. Moreover, the parent DE can also serve as a carbon source for further aerobic degradation [33].

### 3.8. Environmental Relevance

In 2009, a total of 1992 WWTPs treated 2.8 billion m^3^ of wastewater, producing about 20 million tons (80% of water content) of sewage sludge in China [10]. Meanwhile, China is the most important production base of BDE-209 in Asia [34]. The widespread land application of sewage sludge needs to be seriously evaluated in view of the concomitant presence of a variety of contaminants that may negatively affect crops and/or soils [35]. The present study shows that biosorption of aerobic granular sludge contributed to the removal BDE-209 from wastewater significantly. Over 90% of the PBDEs entering a WWTP will ultimately reside within the sludge, with the remainder released via its effluent [5, 6]. We also found that no lower PBDE congener was detected by gas chromatography mass spectrometry during the BDE-209 experiments, indicating that no microbial products were produced. But we observed the biodegradation of lower BDE congeners. Other researchers found that minimal BDE-209 debromination occurred during wastewater treatment and in WWTPs sludge [4]. Although abiotic sorption may limit the potential for human exposure to PBDEs in soil, plants may increase the exposure risk by taking up and translocating PBDEs into tissues and by enhancing bioavailability in soil [36]. Sludge must be treated to reduce odor and pathogen content before application and the metal burden in the sludge is also regulated. But less attention has focused on persistent organic pollutants, such as PBDEs. There are few standards regarding PBDE-contaminated land. The US EPA standards for penta-BDE and deca-BDE in residential soil are 120 and 610 mg/kg; however these standards do not consider potential human health effects [37]. Human health protection calls for standards or guidelines for PBDEs in different land uses (agricultural, playgrounds, and commercial). At the same time the current study warns us to reconsider the application of PBDE-laden biomass solids as fertilizers and soil amendments.

## 4. Conclusions

The biosorption and biodegradation of PBDEs by aerobic granules were examined. The biosorption of BDE-209 was highly dependent on the solution pH, ionic strength, sludge dosage, initial BDE-209 concentrations, and temperature. No biodegradation of BDE-209 occurred in aerobic granular sludge system. BDE-15 disappeared with the production of BDE-3 and DE and the release of bromide. This study calls for standards or guidelines for PBDEs which are specific to different land uses and encourages us to reconsider the land application of PBDEs-laden biomass.

## Supplementary Material

The Supplementary Material contains: pseudo first-order kinetic, modified pseudo first-order kinetic, pseudo second-order kinetic, and intraparticle diffusion models along with Arrhenius equation.

## Figures and Tables

**Table 1:** Effect of ionic strength on bioremoval of BDE-209 by aerobic granules.

Ionic strength (M)	*C/C*_* 0*_	
	NaCl	MgCl_2_
0	0.49	0.49
0.1	0.51	0.67
0.5	0.70	0.82

**Table 2:** Kinetic parameters for adsorption rate expressions.

*C*_0_ (mg/L)	*q*_*e*,exp⁡_^a^ (mg/g)	Pseudo first-order model			Modified pseudo first-order model			Pseudo second-order model			Intraparticle diffusion model	
		*q*_*e*,cal_^b^ (mg/g)	*k*_1_ (1/min)	*R* ^2^	*q*_*e*,cal_^b^ (mg/g)	*K*_1_ (1/min)	*R* ^2^	*q*_*e*,cal_^b^ (mg/g)	*k*_2_ (g/mg*·*min)	*R* ^2^	*k*_*p*_ (mg/g·min^1/2^)	*R* ^2^
1	0.133	0.119	0.004	0.904	0.133	0.001	0.993	0.0973	0.169	0.962	0.004	0.964
2	0.318	0.322	0.019	0.986	0.308	0.0007	0.957	0.200	0.146	0.978	0.009	0.926
4	0.685	0.617	0.021	0.988	0.679	0.003	0.971	0.485	0.066	0.986	0.020	0.924

^a^ *q*_*e*,exp⁡_ represents experimental value.

^b^ *q*_*e*,cal_ denotes calculated value.

**Table 3:** Band assignments for FTIR spectral features.

Wavenumber (cm^−1^)	Assignment and vibration type	Surface functional groups
3200–3600	–OH and –NH overlapping stretching vibration	Polymeric compounds and amine
3400–3550	–OH sharp stretching vibration	Polymeric compounds and amine
2925	–CH_2_ asymmetric stretching vibration	
2852	–CH_2_ symmetric stretching vibration	
2471	–OH stretching vibration	
1638	C=O and C–N (amide I) stretching vibration	Protein (peptidic bond)
1550–1450	C–N stretching vibration and N–H (amide II) deformation vibration	Protein
	C–OH and –CH_3_, –CH_2_ stretching vibration	
1483	C–C stretching vibration	
1385	–COO^−^ stretching vibration	Amino acids
1066	C–N stretching	Carbohydrates and nucleic acids
1030	C–OH, –CH_2_ stretching vibration	Phosphorylated proteins and associated alcohols
1200–1000	C–O stretching	Carbohydrates and nucleic acids
<1000	“Fingerprint” zone	Phosphate or sulphur functional groups
900–600	Ring vibration	Aromatic amino acids and nucleotides.
874, 763	C–C in plane bending	
698	Ring in plane bending	
