# Supplementary material for: Interaction of Polybrominated Diphenyl Ethers and Aerobic Granular Sludge: Biosorption and Microbial Degradation
Source: Biomed Res Int. 2014 May 29;2014:274620. doi: 10.1155/2014/274620 (PMC4058842; doi:10.1155/2014/274620)
Supplement: Supplementary file 1 — The Supplementary Material contains: pseudo first-order kinetic, modified pseudo first-order kinetic, pseudo second-order kinetic, and intraparticle diffusion models along with Arrhenius equation. [file 274620.f1.docx]

**Supporting Information**

**Interaction of polybrominated diphenyl ethers and aerobic granular sludge: Biosorption and microbial degradation**

Shou-Qing Ni ^a,^***, Qingjie Cui ^b^, Zhen Zheng ^a^

*^a^ Shandong Provincial Key Laboratory of Water Pollution Control and Resource Reuse, School of Environmental Science and Engineering, Shandong University, Jinan 250100, China*

*^b^ Department of Mechanical & Environmental Protection, Shandong Electric Power Engineering Consulting Institute (SDEPCI), Jinan 250013, China.*

**Model S1. Pseudo-first-order kinetic model**

| $\ln\left( q_{e}-q_{t} \right)=lnq_{e}-k_{1}t$ | (S1) |
| --- | --- |

where *q_e_* and *q_t_* (mg/g) are the amounts of PBDEs adsorbed at equilibrium and at any time *t*, respectively, and *k_1_* (1/min) is the rate constant.

**Model S2. Modified pseudo-first-order kinetic model**

| $\frac{q_{t}}{q_{e}}+\ln\left( q_{e}-q_{t} \right)=lnq_{e}-K_{1}t$ | (S2) |
| --- | --- |

where *K_1_* (1/min) is the rate constant of the modified pseudo-first-order model.

**Model S3. Pseudo-second-order kinetic model**

| $\frac{t}{q_{t}}=\frac{1}{k_{2}{q_{e}}^{2}}+\frac{t}{q_{e}}$ | (S3) |
| --- | --- |

where *k_2_* (g/mg·min) is the rate constant of the pseudo-second-order model.

**Model S4. Intra-particle diffusion model**

| $q_{t}=k_{\mathrm{dif}}t^{0.5}+C$ | (S4) |
| --- | --- |

where *k_dif_* is the intra-particle diffusion rate constant (mg/g·min^1/2^), and *C* is a constant.

**Arrhenius equation:**

The relationship between temperature and rate constant can be illustrated as [1]:

| $\ln k_{2}=-{E_{a}}/\mathrm{RT}+\ln A$ | (S5) |
| --- | --- |

where *A* represents the pre-exponential factor; *E_a_* (kJ/mol) denotes the activation energy; *k_2_* (g/(mg·h)) is the pseudo-second-order rate constant; *R* (8.314 J/(mol·K)) denotes the gas constant; *T* (K) is the solution temperature.

**Reference:**

[1] Logan, S.R., 1982. The origin and status of the Arrhenius equation. J. Chem. Educ. 59, 279-281.
